# The importance of reminders and patient preferences to improve inhaler technique in older adults with COPD

**DOI:** 10.3389/fphar.2022.989362

**Published:** 2023-01-04

**Authors:** P. Barnestein-Fonseca, VM. Cotta-Luque, VP. Aguiar-Leiva, J. Leiva-Fernández, Francisco Martos-Crespo, F. Leiva-Fernández

**Affiliations:** ^1^ Research Unit Instituto CUDECA de Estudios e Investigación en Cuidados Paliativos Fundación CUDECA, IBIMA Plataforma BIONAND, Málaga, Spain; ^2^ Multiprofesional Teaching Unit of Community and Family Care Primary Care District Málaga-Guadalhorce Knowledge Management Unit Málaga-Guadalhorce Health District, Andalusian Health Services, IBIMA Plataforma BIONAND, Málaga, Spain; ^3^ UGC Vélez Sur Area Sanitaria Málaga Este-Axarquía, Andalusian Health Services, IBIMA Plataforma BIONAND, Málaga, Spain; ^4^ Department of Pharmacology and Paediatrics, School of Medicine, University of Malaga (UMA)- IBIMA Plataforma BIONAND, Malaga, Spain

**Keywords:** COPD, chronic obstructive pulmonary disease, inhalation techniques, educational interventions, care seeking behaviors, primary care, general practice < setting of care, treatment adherence

## Abstract

**Objectives:** Medication non-adherence in patients with chronic obstructive pulmonary disease is common. The aim is to evaluate the efficacy of two interventions to improve the inhalation technique (IT) in patients with pulmonary disease is common. Also determine optimal IT reminder time and to test the role of preferences in the intervention selection.

**Method:** 726 pulmonary disease in common patients (consecutive sampling) from two trials: 1) TECEPOC-study (patients’ preference trial/comprehensive cohort design) 2) TIEPOC-study (randomised controlled trial). Interventions: intervention-A (ad-hoc leaflet with instructions about correct IT according Spanish Respiratory Society), intervention B (intervention A+ individual training by instructors). Four visits were performed (baseline, 3, 6 and 12 months). Data on IT, sociodemographic and clinical characteristics, quality of life and respiratory drugs were recorded. Analysis under intention to treat principle. Multivariate analysis was conducted to measure the potential modifying factors of improvement in the IT along follow-up.

**Results:** 660 patients (90.9%) did not perform a correct IT at baseline 89.75% with Handihaler, 86.95% with Turbuhaler, 84.75% with Accuhaler and 87.35% with pMDI. At 12 months, 221 patients 29.9% performed correctly the IT; a decrease in the slope of the curve (correct IT) was detected at 3 months follow-up. Intervention B was the most effective in both trials compared to control group or intervention A, regardless of preferences: 1) TECEPOC Study (preference trial): Intervention B *versus* control group, NNT = 3.22 (IC95%, 2.27–5.52); and *versus* Intervention A, NNT = 3.57 (CI95%, 2.41–6.8). Preferences improved 6.7% in the correct IT without statistical significance. 2) TIEPOC Study (randomized controlled trial): Intervention B *versus* control group, NNT = 1.74 (IC95%, 1.47–2.17), and *versus* intervention A, NNT = 3.33 (CI 95%, 2.43–5.55). No differences were measured between Intervention A and control group.

**Conclusion:** Individual training significantly improves IT. Reminders every 3 months are recommended. Preferences do not influence the intervention effectiveness.

## 1 Introduction

Medication adherence is a critical challenge in many places around the world. Patients who take medications for chronic health conditions take only about half of their prescribed doses, regardless of the number of medications they are prescribed, and questions are emerging as to the necessity of the number of medications. Patient’s adherence to long-term therapy averages 50%. Adherence rates in clinical trials may be as high as 70–90%, but in clinical practice, they range from 10 to 40% (World Health Organization, 2015). Older people are more likely to experience multiple chronic conditions simultaneously, which increases the number of medications taken at the same time, a key risk factor for lack of medication adherence.

When considering Chronic Obstructive Pulmonary Disease (COPD), it is important to also consider an additional problem when it comes to medication adherence. In a recent systematic review about barriers and strategies to improve medication adherence composed of 38 studies, researchers found lack of medication adherence in COPD patients ranging from 22 up to 93% with an average of 60% (Bhattarai et al., 2020).

Most of the treatment options available for this disease are delivered by inhalers, and skills in their use are required (Chronic obstructive pulmonary disease in, 2018; Global Iniciative for COPD, 2022).The inhalation technique consists of several linked steps that are specific to each device. For more than 40 years, it has been observed that the incorrect use of inhalers is a common problem throughout the world (Chrystyn et al., 2017; Price et al., 2018; Duarte-De-Araújo et al., 2019; Lindh et al., 2019; Padmanabhan et al., 2019; Melani, 2021; Barnestein-Fonseca et al., 2022). Up to 94% of patients have shown misuse in various clinical studies (Sanchis et al., 2016; Chrystyn et al., 2017; Dhand et al., 2018; Lindh et al., 2019; Rincon-Montaña, 2019; Melani, 2021; Barnestein-Fonseca et al., 2022) and despite the improvement in the devices, errors regarding the correct inhalation technique have not decreased (Melani, 2021; Lindh et al., 2022).

The National Institute for Health and Care Excellence (NICE) and the Global Initiative for Chronic Obstructive Lung Disease (GOLD) guidelines recommend that prior to prescription of a new inhaler for a patient with COPD, the patient should receive training and education in the use of the device. Both guidelines also advise that inhaler technique should be regularly assessed at each clinic visit (Chronic obstructive pulmonary disease in, 2018; Global Iniciative for COPD, 2022). Patient education can be defined as a planned process of activities designed to enable people to improve knowledge, to acquire skills and facilitate voluntary adaption of behaviours in order to restore, maintain and improve health (Lindh et al., 2022). However, the guidelines do not provide standardised information on how to assess and educate patients on the use of inhalers, and in many cases, this information needs to be tailored to the characteristics of the individual patient (Chronic obstructive pulmonary disease in, 2018; Plaza Moral et al., 2018; Global Iniciative for COPD, 2022; Miravitlles et al., 2022). The lack of information about inhaler use in these guidelines highlights a deficiency in the care for patients with COPD.

Incorrect use is associated with an increased risk of acute exacerbation, hospital admission, emergency room visits, and a need for antimicrobials and oral steroids (Kocks et al., 2018; Ahn et al., 2019). However, in the real world, inhaler mishandling and poor adherence are very common, despite the fact that most COPD patients receive education on inhaler use (Ahn et al., 2020; Barnestein-Fonseca et al., 2022). While the efficacy and safety of the various inhaled agents and drug combinations is a mandatory consideration for healthcare providers when choosing appropriate therapy for a patient, the choice of the device is also a vital factor; a factor for which there exist no regulatory preferences and current clinical strategies provide little guidance (Lavorini et al., 2019). The importance of the physician’s knowledge and understanding of device has also been highlighted. The assumption that healthcare professionals can be relied on to provide patient instruction is questioned by several studies, suggesting that the knowledge and skills of those providing instruction are less than optimal. Most studies indicate that only approximately half of healthcare professionals know how to use an inhaler or perform correct technique (Lareau and Hodder, 2012; Price et al., 2018).

Many inhalers are challenging to use and some require up to eight steps (Plaza Moral et al., 2018). For every device, at least three instructions are required to avoid errors or reduce them to less than 10% (Takaku et al., 2017). To acquire the skills needed for using the inhaler devices correctly, healthcare professionals and patients must be adequately educated and trained (Sanchis et al., 2013; Aksu et al., 2016; Klijn et al., 2017; Yoo et al., 2017; Melani, 2021).

Initial instruction is of great importance for the outcome of inhalation therapy. Written instructions alone are insufficient in teaching correct inhalation techniques and regular direct one-on-one instruction is considered essential for patients to achieve correct use of the devices (Sanchis et al., 2013; Aksu et al., 2016; Klijn et al., 2017; Yoo et al., 2017; Lavorini et al., 2019; Ahn et al., 2020; Melani, 2021; Lindh et al., 2022). Each patient should understand how to perform each step (Sanchis et al., 2016; Duarte-De-Araújo et al., 2019; Barnestein-Fonseca et al., 2022; Global Iniciative for COPD, 2022; Lindh et al., 2022), and healthcare professionals should verify the correct use of inhalers by reporting possible errors identified (Chrystyn et al., 2017; Ahn et al., 2020; Melani, 2021; Barnestein-Fonseca et al., 2022) along with its clinical importance (Melani, 2007; Barnestein-Fonseca et al., 2022), in order to develop interventions that lead to optimal control of the disease and design of new inhalers (Aksu et al., 2016; Axtell et al., 2017; Klijn et al., 2017; Yoo et al., 2017; Dhand et al., 2018; Efil et al., 2020; Ozoglu Aytac et al., 2020; Kim et al., 2021; Melani, 2021; Choomuang et al., 2022). The main objective of these two trials is to evaluate the efficacy of two educational interventions to improve the inhalation technique (IT) in patients with COPD, as well as to determine the optimal IT reminder time and to test the role of preferences in the intervention selection.

## 2 Materials and methods

### 2.1 Study design

We performed two consecutive in time clinical trials: 1) the first one was TECEPOC Study, a multicentre patients’ preference open-label trial or comprehensive cohort design (ISRCTN15106246) and 2) the second TIEPOC Study, a multicentre, open-label, randomised controlled trial (ISRCTN60147249).

TECEPOC trial was approved by the Ethical Committees of Distrito Sanitario Málaga (01/03/2007) and Axarquía (13/05/2008); TIEPOC trial was approved by the Ethical Committees of Distrito Sanitario Málaga (21/12/2010). The protocol of both studies has been broadly explained (Leiva-Fernández et al., 2012; Leiva-Fernández et al., 2014).

### 2.2 Participants, recruitment and setting

A total of 726 patients with COPD from fourteen Primary Care Centres (PCC), seven urban and rural centres in each trial, were selected by non-random consecutive sampling method: 465 patients in the TECEPOC study and 261 patients in the TIEPOC study.

The inclusion criteria were as follows: confirmed COPD diagnosis, clinical assistance at primary care centres in the Malaga province, prescription of inhaled therapy and having agreed to take part in the study by giving signed written consent. Exclusion criteria were: other respiratory conditions which are not included in the COPD definition (bronchiectasis, asthma or cystic fibrosis) and cognitive impairment problems (dementia, Alzheimer, Parkinson, cognitive decline). All these criteria were reviewed in the patient’s clinical record.

The sample size in both trials was calculated aiming at detecting a correct inhalation technique percentage difference between groups of 25%, with a statistical power of 80% and a confidence level of 95%, assuming a percentage of expected losses of 40% throughout the follow-up.

Patients were contacted by telephone and invited to participate; they then received an appointment at the PCC. At this first appointment (inclusion visit), patients were given more detailed information about the study, and if they agreed to participate, they signed the written consent form.

In the TECEPOC trial, patients were asked if they had a preference for any of the interventions and based on this, were divided into two groups. Patients without strong preferences for a treatment were randomised (RCT group) using the block randomisation technique which consisted of blocks of three or six patients homogeneously distributed among the three arms of the trial; randomization was applied separately at each study centre. Those patients with strong preferences were given their choice (PPS group). The RCT group resulted in three arms (control -CG-, intervention A -IAR- and intervention B -IBR-), whereas the PPS group ended up with two arms (intervention A -IAP- and intervention B -IBP-), so in the end this study had five arms.

In the TIEPOC trial, patients were directly allocated to one of the three study arms using a block randomization technique, following the same procedure as in the previous trial.

### 2.3 Interventions

Two educational interventions were designed and applied in both trials: 1) Intervention A (IA) that provided only written information about inhalation techniques; and 2) Intervention B (IB) that consisted in written information about inhalation techniques + instructor-led training.

Intervention A (IA): The research team designed a leaflet explaining the correct inhalation techniques, containing the main devices the patients use in our area. We included four devices: Handihaler^®^, Turbuhaler^®^, Accuhaler^®^ and Pressurised Metered Dose Inhalers (pMDI). It was written in simple language so that patients could understand the information, with original photos showing the main steps for each device. The leaflets were designed and written by the research team, after consulting the manufacturer’s instructions and SEPAR recommendations and reviewed by experts (family doctor and pulmonologist). Subsequently, patients were asked to review them and gave feedback on their ease of understanding and use of plain language. The patients included in this group were asked to demonstrate how they used their devices with placebo inhalers, and the researcher wrote down the mistakes on an *ad hoc* template designed according to the Spanish Society of Pneumology and Thoracic Surgery (SEPAR) guidelines (Plaza Moral et al., 2018). Once the inhalation techniques were performed, the researcher gave the leaflet to the patients and invited them to read it and identify differences between the steps of the correct inhalation technique (leaflet) and the ones they had performed. In the follow-up visits, patients were asked about the leaflet and the differences between those instructions and their technique.

Intervention B (IB): The research team gave written information (leaflet described above) to patients and also trained them in correct inhalation techniques. The training was performed by four researchers (instructors) that were trained in the use of inhaler devices in the Paediatric Pneumology Department of the Hospital Materno Infantil (Malaga). First, patients were asked to demonstrate their technique with placebo inhalers. Then, the instructor, using the teach-back method, asked about the problems and perceived errors with the technique and proceeded to demonstrate the proper technique with each device, step by step, including the importance of each one. Finally, patients could ask questions and practice the techniques until they were performed correctly or until the patient became tired. In the follow-up visits, the inhalation technique was reviewed and errors were corrected again and doubts were cleared out. The goal at this stage was to identify errors, and if they could not, to remind them of the proper technique by giving as many demonstrations as necessary.

Patients in the control arm in both trials were asked to demonstrate their technique without any further intervention from the researcher apart from correcting critical errors (rescue mechanism). The critical error has been established as the one that would considerably reduce drug lung deposition (Melani, 2007). There was no leaflet or educational intervention involved.

All patients had four follow-up visits: baseline, 3, 6 and 12-month.

### 2.4 Outcomes

Primary outcome: Performance of correct inhalation techniques following SEPAR guidelines (Plaza Moral et al., 2018) at 12-month follow-up. A correct technique will be considered when no mistakes are registered.

Secondary outcomes: Performance of correct inhalation techniques following SEPAR guidelines (Plaza Moral et al., 2018) at three and 6-month follow-up, inspiratory peak flow, functional status (spirometry:pFEV1 and severity according to GOLD Guidelines (Global Iniciative for COPD, 2022)), dyspnoea measured with Baseline Dyspnoea Index (BDI) (Mahler et al., 1984) and Modified Medical Research Council (MMRC) (Devon and Holman, 1966); Quality of life: St George Respiratory Questionnaire (SGRQ) (Ferrer et al., 1996), and EuroQoL-5D-3L (Herdman et al., 2011).

Independent variables: The following variables were included; age, sex, educational level (considering the highest level of education attained as reported by the patient at the baseline visit), comorbidities (other chronic diseases diagnosed to the patient, according to his/her electronic health record), smoking history (patient-reported smoking habit, considering the options non-smoker, ex-smoker or current smoker, number of packs-year) and Mini-mental State Examination (MMSE) (Lobo et al., 2002).

Related to COPD: prescribed treatment for COPD, time of diagnosis, number of prescribed devices, number of exacerbations, number of visits to the healthcare centre because of COPD, previous instruction received regarding IT, type of instruction and professional who gave it, types of error in the IT and time for inhaler training (including test of the performance of inhalation techniques of all the devices used by the patient).

### 2.5 Statistical analysis

The analysis was carried out following an intention-to-treat procedure, considering all patients who were randomised, irrespective of what happened during follow-up in both studies. A Multivariate Imputation has been used to handle missing data. For the primary outcome variable, the handling of lost data was done using the worst scenario considering that losses in the control group performed the IT correctly and those in the intervention groups performed the IT incorrectly.

A descriptive statistical analysis was performed for all of the study variables. We calculated the mean and standard deviations for quantitative variables and the absolute and relative frequencies for qualitative variables. Univariate analyses: a between-group comparison at baseline, a comparison between the initial sample and the final sample (to assess the impact of losses on sample structure), a comparison between each intervention arm (A or B) *versus* control arm and between intervention A and B at 12-month follow-up was conducted by means of an analysis of variance (ANOVA) or chi square test, as applicable. The relative risk reduction (RRR), the absolute risk reduction (ARR) and the number needed to treat (NNT) were calculated with a CI of 95%. Multivariate analyses: a logistic regression model was performed for the primary outcome (performance of correct inhalation technique at 12-month), considering the intervention as the predictive variable and adjusting for independent variables that may act as modifying factors of the effect of the intervention.

In the case of the TECEPOC trial, due to its special design, each group (RCT and PPS) was analysed separately. The analysis has been performed according to the following steps: 1) Comparison in RCT group: each intervention arm (A or B) *versus* control arm and between intervention A and B. 2) Comparison between RCT and PPS groups: between the intervention arms (A or B) of each group (RCT or PPS). An analysis of variance (ANOVA) or ji-squared test were applied as stated above.

We used a 5% significance level (α = 0.05) and the SPSS statistical package, version 23.0, to run the proposed analysis.

## 3 Results

For clarity purposes, both trials will be detailed separately in this section, as they were conducted at consecutive times and in different primary care centres. The findings regarding inhalation technique are described in a unique paragraph so as to be more instructive.

### 3.1 Participant recruitment

**FIGURE 1:**
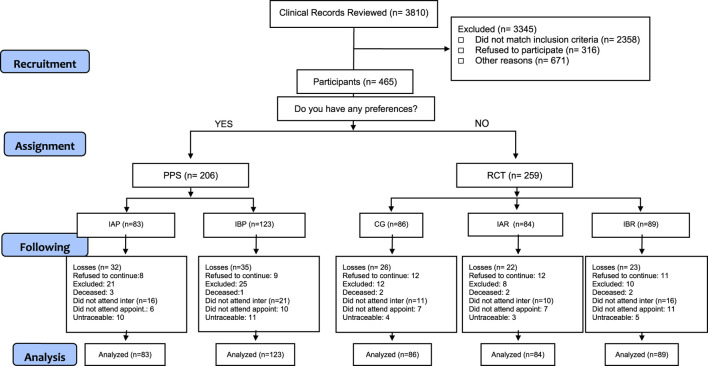
Consort flow tecepoc study.

**FIGURE 2:**
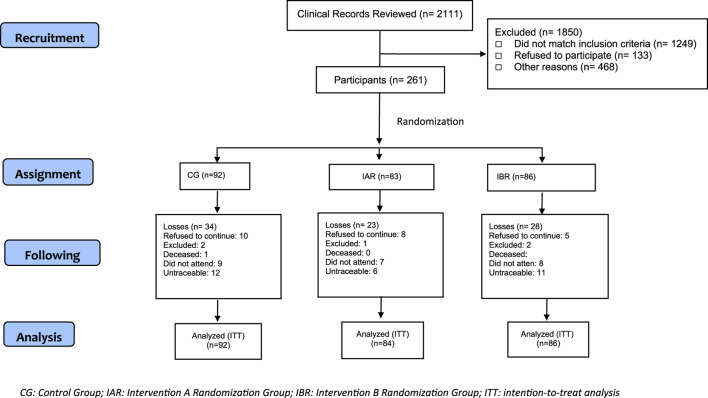
Consort Flow tecepoc study.

For both the TECEPOC Study and the TIEPOC Study we approached 5,921 potential participants identified in clinical records. At the end, 726 patients were recruited to participate, 465 in the TECEPOC Study and 261 in the TIEPOC Study. Figures 1, 2 show the CONSORT Flow Diagram of both studies.

### 3.2 Follow-up

In the TECEPOC Study, 97 patients were lost to follow-up (dropout rate 20.86%): 40 patients (19.41%) in the PPS group and 57 (22%) in the RCT group. For the TIEPOC Study the dropout rate was 30.3%, which corresponds to 79 patients: 35 (38%) in the CG, 21 (25.3%) in the IAR and 21 (25.3%) in the IBR. Figures 1, 2 show the CONSORT Flow Diagram of both studies.

These losses did not change the initial characteristics of the sample for the TECEPOC Study. For the TIEPOC Study statistically differences in the final sample were found for sex (higher dropout rate among women; *p* = 0.021), age (older participants missed more; *p* = 0.005) and cognitive status (more dropouts in participants with lower MMSE scores; *p* = 0.018).

### 3.3 Baseline characteristic

In Table 1, we can see the baseline characteristic of participants per study arms.

**TABLE 1:** Descriptive of the variables at baseline according to the study arm.

	**TECEPOC Study (n=465)**					**TIEPOC Study (n=261)**		
	**PPS (n=206)**		**RCT (n=259)**			**RCT (n=261)**		
**Variables**	IAP (n=83)	IBP (n=123)	CG (n=86)	IAR (n=84)	IBR (n=89)	CG (n=92)	IAR (n=83)	IBR (n=86)
Sex n(%) Male	81 (96.4)*	116 (94.3)*	75 (87.2)*	74 (89.3)*	79 (88.8)*	83 (90.2)	72 (86.74%)	72 (83.72)
Age (years) mean (CI 95%)	70.1 (68.3-71.9)	69.6 (68.2-71)	70.2 (68.4-72.1)	68.4 (66.4-70.4)	70.5 (68.5-72.5)	70.11 (68.45-71.76)	70.05 (68.01-72.09)	69.99 (68.09-71.89)
Low educational level n (%)	75 (92.5)*	112 (91.9)*	65 (76.5)*	69 (83.3)*	76 (85.4)*	64 (69.56)	56 (70)	64 (75.3)
Smokers n (%) Packets/year men (CI 95%)	31 (36.9) 56.3 (44.5-68.1)	35 (28.5) 61.2 (52.6-69.8)	23 (26.7) 52.1 (42.6-61.7)	23 (27.4) 57.65 (47-68.3)	28.1 66.9 (56.2-77.5)	26 (28.26) 58.42 (50.28-66.56)	32 (38.55) 51.98 (43.17-60.79)	22 (25.0) 46.11 (37.01-55.2)
Comorbidities								
• Number	0.89 (0.79-1)	0.94 (0.83-1.06)	0.97 (0.89-1.06)	1.12 (1.01-1.23)	1.03 (0.93-1.14)	1.10 (0.94-1.27)	0.9 (.071-1.09)	0.93 (0.76-1.09)
• HBP n (%)	42 (50.6)	62 (50.4)	43 (50)	43 (51.2)	40 (44.9)	52 (56.52)*	30 (36.14)*	43 (50)*
• OP n (%)	18 (21.7)*	27 (22)*	29 (33.7)*	32 (38.1)*	58 (34.8)*	23 (25)	24 (28.91)	18 (20.93)
• DM n (%)	15 (18.1)	27 (22)	12 (14)	18 (21.4)	68 (23.6)	27 (29.34)	21 (25.3)	19 (22.09)
Diagnostic time (years) mean (CI 95%)	6.1 (5-7.3)	6.7 (5.6-7.9)	6.3 (5-7.7)	5.3 (4.4-6.2)	6.6 (5.7-7.6)	10.92 (8.24-13.61)	8.42 (6.2-10.63)	9.91 (8.12-11.69)
COPD pattern n (%)								
• Obstructive	5 (6.3)	13 (11.1)	25 (29.6)	22 (27.5)	28 (32.9)	29 (31.5)	24 (28.9)	20 (23.3)
• Restrictive	12 (15)	16 (13.7)	9 (11.1)	12 (15)	7 (8.2)	2 (2.2)	8 (9.6)	4 (4.7)
• Mixed	64 (78.8)*	88 (75.2)*	46 (56.8)	45 (57.5)	49 (57.6)	46 (50)	39 (47)	47 (54.7)
COPD severity n (%)								
• Mild	7 (8.8)	9 (7.7)	19 (24.4)	14 (17.3)	20 (23)	13 (16.3)	10 (13.7)	5 (6.8)
• Moderate	31 (38.8)	53 (45.3)	35 (42.7)	43 (53.1)	35 (40.2)*	45 (56.3)	39 (53.4)	32 (43.2)
• Severe	43 (52.5)*	55 (47)*	28 (32.9)	23 (29.6)	32 (36.8)	22 (27.5)	24 (32.9)	37 (50)
FEV1 % (CI 95%)	49.07 (46.64-51.5)*	52.48 (50-54.97)*	60.3 (57.47-63.13)*	58.17 (55.88-60.46)*	56.78 (54.35-59.21)*	61.03 (56.61-65.46)*	59.01 (55.28-62.74)*	52.19 (48.36-56.02)*
Inspiratory peak flow (CI 95%)	155.88 (148.6-163.1)*	165.38 (158.5-172.1)*	173.41 (165.4-181.3)*	181.46 (174.1-188.8)*	174.12 (166.7-181.5)*	186.85 (178.1-195.6)	192.53 (184.3-200.6)	188.29 (180.3-196.2)
Number of exacerbations/year mean (CI 95%)	0.3 (0.2-0.5)*	0.8 (0.6-1)*	1.2 (0.8-1.5)*	0.7 (0.5-0.91)*	1.8 (0.6-1.4)*	0.93 (0.57-1.3)	0.71 (0.48-0.93)	0.8 (0.58-1.02)
Total visits to HC (CI 95%)	5.76 (5.03-6.49)*	4.97 (4.34-4.59)*	7.36 (6.46-8.26)*	6.43 (5.68-7.18)*	6.4 (5.73-7.07)*	5.67 (5.17-6.17)	6.48 (5.79-7.17)	5.36 (4.83-5.89)
Visits to HC because of COPD (CI 95%)	1.3 (0.9-1.6)*	1.7 (1.3-2.1)*	3 (1.6-4.4)*	1.7 (1.3-2.1)*	1.95 (1.4-2.4)*	1.68 (1.29-2.06)	1.64 (0.82-2.46)	1.61 (1.17-2.04)
Prescribed treatment n (%)								
• Anticholinergic	64 (76.8)	90 (73.2)	61 (70.9)	56 (67.9)	57 (64)	75 (81.52)	17 (79.51)	69 (80.2)
• Beta-2 adrenergic	67 (80.5)*	115 (93.5)*	76 (88.4)	74 (89.3)	80 (89.9)	78 (84.78)	72 (86.74)	69 (80.2)
• Inhaled corticosteroids	50 (70.2)	99 (80.5)	66 (76.7)	60 (72.6)	72 (80.9)	73 (79.3)	61 (73.5)	63 (73.3)
SGRQ mean (CI 95%)								
• Total	34.8 (30.6-39)*	34.6 (30.6-39)*	33.4 (29.4-37.3)	31 (27.2-34.8)	33 (29.3-36.6)	33.7 (30.2-37.3)	33.1 (29.9-36.3)	34.7 31.8-37.6)
• Activities	55.8 (50.8-60.8)*	54.3 (50.1-58.5)*	49.6 (44.6-54.6)	49.1 (44.4-53.8)	49.9 (45-54.7)	49.44 (44.9-53.9)	47.9 (43.6-52.)1	52.7 (48.6-56.8)
•Symptoms	35.2 (30.5-39.9)	36.8 (33.3-40.3)	36.9 (32.5-41.3)	34.8 (30.6-39.1)	36.1 (32.1-40.2)	35 (30.8-39.2)	35.7 (31.5-39.9)	36.7 (32.8-40.7)
• Impact	23.1 (19-27.4)	22.7 (19.3-26.1)	25.3 (21.3-29.3)	22.5 (18.9-26.1)	24.4 (20.6-28.1)	24.2 (20.4-28.1)	23.8 (20.5-27.1)	23.7 (20.7-26.7)
EuroQol-5D n (%) with no problems								
• Mobility	67 (80.5)	88 (71.5)	61 (70.9)	51 (61.9)	54 (61.4)	54 (60)	48 (57.83)	54 (62.79)
• Self-care	74 (89)	104 (84.6)	80 (93)	73 (88.1)	76 (88.6)	80 (87.91)	75 (90.36)	81 (94.18)
• Usual activities	65 (78)	105 (85.4)	75 (87.2)	73 (88.1)	76 (86.4)	78 (85.71)	71 (85.54)	76 (88.37)
• Anxiety/depression	62 (74.4)	91 (74)	64 (74.4)	59 (71.4)	65 (73.9)	70 (76.92)	66 (79.51)	65 (75.58)
• Pain/discomfort	65 (78)*	89 (72.4)*	57 (54.7)*	45 (54.8)*	47 (53.4)*	56 (61.53)	42 (60.6)	50 (58.13)
• EVA	64.98 (62.6-67.3)	68.33 (65.6-71.0)	66.34 (63.8-68.8)	67.65 (65.1-70.1)	64.94 (62.6-67.2)	67.2 (63.1-71.3)	65.7 (61.2-70.1)	67.9 (64.2-71.6)
BDI n (%)								
• Functional Impairment	52 (62.7)	76 (62.3)	53 (62.4)	48 (57.8)	48 (55.2)	31 (34.44)	34 (40.96)	39 (45.34)
• Magnitude of task	63 (75.9)	93 (76.2)	64 (76.2)	65 (78.3)	64 (74.4)	62 (68.89)	60 (72.28)	71 (82.55)
• Magnitude of effort	78 (94)	91 (74.6)	66 (77.6)	67 (80.7)	71 (82.6)	65 (72.23)	59 (71.08)	73 (84.88)
MMRC n (%)	78 (94)	118 (96.7)	77 (92.8)	79 (95.2)	75 (87.2)	40 (44.45)	35 (42.16)	44 (51.16)
MMSE mean (CI95%)	26.5 (25.8-27.1)	26.4 (25.9-27)	26.7 (26.2-27.2)	26.6 (26-27.2)	26.6 (26-27.2)	28.16 (27.7-28.6)	28.37 (27.9-28.9)	28.1 (27.5-28.6)

^*^ *p* < 0.05; BMI: body mass index; CG: control group; DM: diabetes mellitus; HBP: high blood pressure; HC: health center; IAP: Intervention A Cohort Preference Group; IAR: Intervention A Cohort Randomization Group; IBP: Intervention B Cohort Preference Group; IBR: Intervention B Cohort Randomization Group; MMST: Mini-Mental Status Test; OP: osteoarticular pathology; PPS: patient preferences group; RCT: randomized group; SGRQ: St. george respiratory questionnaire; *: statistically significant differences (*p* < 0.05).

Overall, the 465 subjects of TECEPOC Study were predominantly male (91.4%), with a mean age of 69.8 years (95% CI, 69.41–70.19) with low educational level; most of them had smoked (92.9%) with a mean of 39.78 packs per year (95% CI, 39.24–40.32), and 29.5% were active smokers. A large part of the sample suffered from at least one additional chronic condition, most prevalent was high blood pressure (HBP) (49.5%); with a moderate impairment of quality of life. Regarding COPD, the spirometry revealed a mean pFEV1 of 55% (95% CI, 52.71–57.37), with a mixed pattern (65.9%), and a mean of 0.83 exacerbations in the previous year (95% CI, 0.72–0.94) (Table 1).

Overall, the 261 subjects in the TIEPOC study were very similar to those in the TECEPOC study, showing a majority of male (86.97%), with a mean age of 70.17 years (95% CI, 69–71.1 years), and low educational level; most of them had smoked (91.95%) with a mean of 52.32 packs per year (95% CI, 47.36–57.27), and 30.7% were active smokers. A large part of the study subjects suffered from at least one additional chronic condition, most prevalent was HBP (47.29%), with a moderate impairment of quality of life. Regarding COPD, the spirometry revealed a mean FEV1 of 57.47% (95% CI, 55.32–59.62), with a mixed pattern, and a mean of 0.82 exacerbations in the previous year (95% CI, 0.66–0.98) (Table 1).

No significant differences were observed between the arms in the RCT group of the TECEPOC study, but significant differences were found between the arms of the PPS group in relation to number of exacerbations (*p* = 0.004), beta two adrenergic treatment (more at IBP; *p* = 0.005) and Accuhaler^®^ prescription (more at IBP; *p* = 0.049). We also found significant differences between PPS and RCT group: there were low values in PPS group related to: number of women (*p* = 0.01), educational level (*p* = 0.002), osteoarthritis comorbidity (*p* = 0.001), pFEV1 (*p* < 0.001), number of exacerbations (*p* = 0.012), number of total visits to health centre (*p* = 0.008) or due to COPD (*p* = 0.036), peak flow (*p* = 0.048) and pain/discomfort problems on the EuroQol-5D scale (*p* < 0.001). There were higher values in the PPS group in COPD severity (high percentage of severe stage; *p* < 0.001) and mixed pattern (*p* = 0.004). Also, we found high impairment in health-related quality of life measured by the activity scale of SGRQ (*p* = 0.012).

For the TIEPOC Study we found significant differences for HBP (IAR cohort showed lower prevalence; *p* = 0.024) and for pFEV1 value (IBR cohort had lower pFEV1; *p* = 0.006).

Considering the total number of patients between the two studies, 660 patients (90.9%) did not perform a correct inhalation technique at baseline. The device Handihaler® was prescribed in 508 (69.97%), the Turbuhaler® in 396 (54.54%), 235 with the Accuhaler® (32.36%), 178 with the pMDI (24.51%) and 101 patients with other devices (13.9%). Incorrect inhalation technique was detected in 456 subjects (89.75%) with Handihaler®, 340 (86.95%) with Turbuhaler®, 198 (84.75%) with Accuhaler® and 143 (87.35%) with pMDI.

Six hundred and fourteen patients (84.57%) had received some kind of inhaler technique instruction and the mean time from this instruction to recruitment in the present studies was 55.48 months (95%CI, 46.17–55.11). Previous instruction was performed mainly by the pulmonologist (294 patients; 47.88%), followed by the family physician (248 patients; 40.39%). The most common method used to carry out this instruction was the device-less explanation (346 subjects; 56.35%), followed by demonstration with the device (137 subjects; 22.31%). In six patients (0.9%) the instruction consisted on the delivery of an explanatory leaflet.

The most frequent errors identified were: 1) not exhaling completely before inhaling (76.4%), 2) no breath-holding or shortness of breath after inhalation (64.21%), and 3) a non-optimal strength of inhalation (20.32%). The more frequent mistakes related to the devices were: the coordination of breath for pMDI (57.3%) and position of the device (hold inhaler upright >45^o^) for Turbuhaler^®^ (92.21%).

Table 2 collects the baseline characteristics of the inhalation technique in both studies.

**TABLE 2:** Descriptive of the Inhalation Technique at baseline according to the study arm.

	*TECEPOC Study*					*TIEPOC Study*		
	*PPS*		*RCT*			*RCT*		
***Variables***	*IAP*	*IBP*	*CG*	*IAR*	*IBR*	*CG*	*IAR*	*IBR*
*Correct Inhalation Technique n%*	*7 (8.4)*	*4 (3.3)*	*10 (11.6)*	*6 (7.1)*	*6 (6.7)*	*9 (9.7)*	*13 (15.7)*	*11 (12.8)*
*Number of devices mean (CI 95%)*	*2.02 (1.8-2.2)*	*2.05 (1.9-2.1)*	*2.09 (1.9-2.2)*	*2.06 (1.8-2.2)*	*2.07 (1.9-2.2)*	*2.15 (1.95-2.35)*	*2.12 (1.93-2.31)*	*2.03 (1.85-2.22)*
*Prescribed devices n (%)*								
• *Handihaler©*	*61 (72.6)*	*85 (69.1)*	*59 (68.6)*	*54 (65.1)*	*54 (60.7)*	*73 (79.3)*	*60 (72.3)*	*62 (72.1)*
• *Accuhaler©*	*20 (23.8)* *	*44 (35.8)* *	*31 (36)*	*27 (32.5)*	*26 (29.2)*	*29 (31.5)*	*32 (38.6)*	*26 (30.2)*
• *Turbuhaler©*	*49 (58.3)*	*66 (53.7)*	*41 (47.7)*	*46 (55.4)*	*53 (59.6)*	*58 (63)*	*45 (54.2)*	*34 (44.2)*
• *pMDI*	*20 (23.8)*	*28 (22.8)*	*29 (33.7)*	*23 (27.7)*	*25 (28.1)*	*15 (16.3)*	*19 (22.9)*	*19 (22.1)*
***Handihaler***								
• *Correct Inhalation Technique n (%)*	*6 (7.1)*	*5 (4.1)*	*7 (8.1)*	*5 (6)*	*4 (4.5)*	*7 (7.6)*	*9 (10.8)*	*7 (8.1)*
*Mistakes*								
• *No full exhale before inhalation n (%)*	*7 (8.3)*	*12 (9.8)*	*14 (16.3)*	*8 (9.6)*	*6 (6.7)*	*15 (16.3)*	*16 (19.4)*	*12 (14)*
• *No or short breath hold after inhalation n (%)*	*11 (13.1)*	*13 (10.6)*	*19 (22.1)*	*18 (21.7)*	*18 (20.2)*	*28 (30.4)*	*21 (25.3)*	*23 (26.7)*
• *Non-optimal strength of inhalation n (%)*	*58 (69)*	*78 (63.4)*	*54 (62.8)*	*47 (56.6)*	*47 (52.8)*	*58.7 (54)*	*54 (65.1)*	*48 (55.8)*
***Accuhaler***								
• *Correct Inhalation Technique n (%)*	*3 (3.6)*	*6 (4.9)*	*5 (5.8)*	*4 (4.8)*	*3 (3.4)*	*5 (5.4)*	*6 (7.2)*	*3 (3.5)*
*Mistakes:*								
• *No full exhale before inhalation n (%)*	*10 (11.9)*	*29 (23.6)*	*9 (10.5)*	*4 (4.8)*	*8 (9)*	*8 (8.7)*	*11 (13.3)*	*3 (3.5)*
• *No or short breath hold after inhalation n (%)*	*5 (6)*	*15 (12.2)*	*14 (16.3)*	*13 (15.7)*	*24 (27)*	*10 (10.9)*	*14 (16.9)*	*16 (18.6)*
• *Non-optimal strength of inhalation n (%)*	*16 (19)*	*40 (32.5)*	*28 (32.6)*	*23 (22.7)*	*1 (1.1)*	*22 (23.9)*	*30.1 (25)*	*18 (20.9)*
***Turbuhaler***								
• *Correct Inhalation Technique n (%)*	*6 (7.1)*	*3 (2.4)*	*9 (10.5)*	*4 (4.8)*	*4 (4.5)*	*7 (7.6)*	*8 (9.6)*	*7 (8.1)*
*Mistakes:*								
• *No full exhale before inhalation n (%)*	*7 (8.3)*	*11 (8.9)*	*14 (16.3)*	*6 (7.2)*	*11 (12.4)*	*14 (15.2)*	*13 (15.7)*	*9 (10.5)*
• *Not placing lips correctly on the mouthpiece n (%)*	*47 (56)*	*62 (50.4)*	*40 (46.5)*	*44 (53)*	*52 (58.4)*	*56 (60.9)*	*44 (53)*	*36 (41.9)*
• *No or short breath hold after inhalation n (%)*	*9 (10.7)*	*14 (11.4)*	*14 (16.3)*	*17 (20.5)*	*13 (14.6)*	*22 (23.9)*	*17 (20.5)*	*15 (17.4)*
• *Non-optimal strength of inhalation n (%)*	*43 (51.2)*	*58 (47.2)*	*37 (43)*	*42 (50.6)*	*50 (56.2)*	*48 (52.2)*	*37 (44.6)*	*34 (39.5)*
***pMDI***								
• *Correct Inhalation Technique n (%)*	*1 (1.2)*	*21 (17.1)*	*2 (2.3)*	*3 (3.6)*	*2 (2.2)*	*3 (3.3)*	*1 (1.2)*	*5 (5.8)*
*Mistakes:*								
• *No full exhale before inhalation n (%)*	*1 (1.2)*	*6 (4.9)*	*8 (9.3)*	*5 (6)*	*8 (9)*	*4 (4.3)*	*6 (7.2)*	*8 (9.3)*
• *No or short breath hold after inhalation n (%)*	*3 (3.6)*	*8 (6.5)*	*5 (5.8)*	*6 (7.2)*	*12 (13.5)*	*10 (10.9)*	*4.8 (4)*	*11 (12.8)*
• *No coordination after push n (%)*	*14 (16.7)*	*18 (14.6)*	*17 (19.8)*	*9 (10.8)*	*16 (18)*	*10 (10.9)*	*8 (9.6)*	*10 (11.6)*
• *Non-optimal strength of inhalation n (%)*	*9 (10.7)*	*12 (9.8)*	*14 (16.3)*	*4 (4.8)*	*12 (13.5)*	*6 (6.5)*	*6 (7.2)*	*10 (11.6)*

^*^ *p* < 0.05; CG: control group; IAP: Intervention A Cohort Preference Group; IAR: Intervention A Cohort Randomization Group; IBP: Intervention B Cohort Preference Group; IBR: Intervention B Cohort Randomization Group; PPS: patient preferences group; RCT: Randomized group.

### 3.4 Intervention effectiveness

**FIGURE 3:**
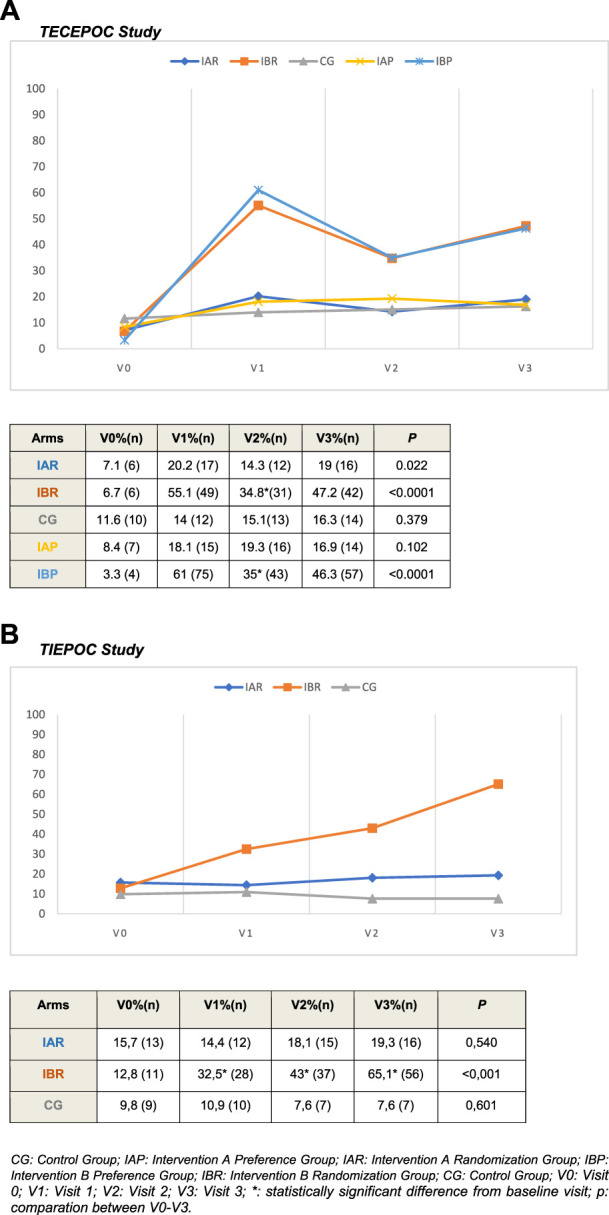
Correct inhalation technique during follow-up according to study arms.

Figure 3 shows the evolution of inhalation techniques along follow up (Figure 3A the five arms of TECEPOC Study and Figure 3B the three arms of TIEPOC Study).

About time for inhaler training, it was 5.19 min (IC95%, 4.91–5.47) for CG cohort, 6.2 min (IC95%, 5.74–6.5) for IAR cohort and 7.15 min (6.8–7.5) for IBR cohort at baseline. At the end of the study it was 3.68 min (IC95%, 3.38–3.98) for CG cohort, 4.02 min (IC95%, 3.75–4.29) for IAR cohort and 4.18 min (IC95%, 3.89–4.47) for IBR cohort.

### 3.4.1 TECEPOC study

At the end of study, the correct inhalation techniques in the RCT group were: 16 (19%) patients for IAR, 42 (47.2%) patients for IBR cohort and 14 (16.3%) patients for CG cohort. There were no differences between CG and IAR cohorts. There were statistically significant differences between IBR cohort *versus* CG cohort in all the follow-up visits (*p* < 0.0001); and at the end of study the NNT for IBR was 3.22 (CI 95%, 2.27–5.52). In the same way, there were significant differences at 12 months between IBR *versus* IAR (*p* < 0.0001) with a NNT = 3.57 (CI95%, 2.41–6.8).

For the PPS group the correct inhalation technique at the end of follow-up was assessed in 14 patients (16.9%) for the IAP cohort and in 57 patients (46.3%) for the IBP cohort. Statistically significant differences (*p* < 0.0001) were found between the IB cohort *versus* IAP cohort with a NNT = 3.33 (CI 95%, 2.43–5.55).

Inhalation techniques at 3 and 6 months (as secondary results) showed a statistically significant improvement in the two IB cohorts (*p* < 0.0001). A decrease in the slope of the curve (correct IT) was detected at 3 months of follow-up in both IB cohorts. There were no differences between CG and IA cohorts.

For the other secondary outcomes, we found better results in all study arms at the end of the study (respect to baseline measurement) for inspiratory peak flow (*p* = 0.001), anxiety/depression scale of EuroQoL-5D (*p* < 0.0001), SGRQ for symptom scale (*p* = 0.016), activity scale (*p* < 0.0001) and total scale (*p* = 0.005). In the same way we detected an improvement in all scales of IBD with less perceived dyspnoea (*p* < 0.0001).

### 3.4.2 TIEPOC study

At the end of study, the percentages of correct inhalation techniques were: 16 patients (19.3%) for IAR, 56 patients (65.1%) for the IBR cohort and seven patients (7.6%) for the CG cohort. There was no difference between CG and IAR cohorts. There were statistically significant differences between the IBR cohort and CG in all the follow-up visits (*p* < 0.0001), with a NNT of 1.74 patients (IC 95%, 1.47–2.17) at the end of the study.

Inhalation techniques at 3 and 6 months (as secondary results) showed a statistically significant improvement in the IBR cohort (*p* < 0.0001). As in the previous study, a change in the slope of the correct inhalation technique curve was detected at 3-month follow-up.

For the other secondary outcomes, we found better results in all study arms at the end of follow-up (respect to baseline) for severity (*p* = 0.003), number of exacerbations (*p* < 0.0001), SGRQ for all its scales symptom, activity, impact and total scale (*p* < 0.0001). In the same way we detected an improvement in all scales of MMRC with less perceived dyspnoea (*p* < 0.0001).

### 3.5 Preferences effects

Preferences regarding study group assignment were associated with an increase in the percentage of correct inhalation technique of 6.7% in the IBP cohort at 3-month follow-up which was reduced to 1% at the end of the study. For the IAP cohort, preferences are associated with a 2% improvement in inhaler technique at 12 months. None of these changes showed statistical significance.

### 3.6 Multivariate analysis

#### 3.6.1 TECEPOC study

We performed a logistic regression model considering the correct inhalation technique as the dependent variable and the intervention as the predictive variable, adjusting by preferences, age, sex, educational level, number of exacerbations and inspiratory peak flow, functional status, number of devices, health related quality of life measurements and MMSE. The final logit model showed that correct inhalation technique was positively associated with the IB [OR = 31.5 (CI 95% 8.273–50.9) *p* < 0.0001], higher inspiratory peak flow [OR = 1.010 (CI 95%, 1.003–1.017) *p* = 0.007], higher number of devices [OR = 2.615 (CI 95%, 1.473–4.645) *p* = 0.001] and previous instruction with device demonstration [OR = 3.54 (CI 95%, 1.38–9.07) *p* = 0.008]. The correct inhalation technique got worse in patients with lower SGRQ activity scale score [OR = 0.975 (CI 95%, 0.956–0.90) *p* = 0.015].

#### 3.6.2 TIEPOC study

We performed a logistic regression model considering the correct inhalation technique as the dependent variable and the intervention as the predictive variable, adjusting by age, sex, educational level, number of comorbidities, HBP, inspiratory peak flow, number of exacerbations, functional status, number of devices, SGRQ scales and MMSE. The final logit model showed that correct inhalation technique was positively associated with the IB [OR = 26.34 (CI 95% 10.42–66.57) *p* < 0.0001] and it worsened in older patients [OR = 0.934 (CI 95%, 0.89–0.97) *p* = 0.001].

## 4 Discussion

The TECEPOC and TIEPOC studies assessed, as primary outcome, the correct performance of inhalation technique and the efficacy of the same two educational interventions to improve the inhalation technique in patients with COPD. We found the most effective intervention to be the one-to-one demonstration of inhaler use with application of the teach-back method, while the provision of an information leaflet resulted in an improvement in inhaler technique close to that of the control group. The evolution of the improvement in inhaler technique over the follow-up showed that the upward trend in the proportion of patients who could use the devices correctly slowed down 3 months after the training.

Proper training can improve inhaler technique (Klijn et al., 2017). However, there are several different levels of education and related to these levels there are different teaching techniques. Basically, we can divide those teaching techniques into two groups: leaflets and practical demonstration.

A systematic review of educational inhaler technique interventions (Klijn et al., 2017) showed that almost all interventions (89%) included a physical or video demonstration of inhaler use and that the educational interventions on inhaler technique are effective, at least in the short term. All studies showed improvements and statistical significance with a mean intervention time of 30 min and an average follow-up of 5 months. Whether or not patients were requested to demonstrate their own inhaler use after demonstration was frequently not reported. Approximately half of the studies provided additional disease education or embedded the inhaler education in a more complex intervention. Another one that collects the interventions based on the Information-Motivation-Behavioural skills (IMB model) showed that these interventions based on the demonstration of inhalation technique may be more effective (Jia et al., 2020).

When looking at studies that evaluate both types of educational interventions together, we found that Bosnic-Anticevich et al. (Bosnic-Anticevich et al., 2010) referred to an improvement of 89% for the group receiving the demonstration, opposed to an improvement of 44% for the group receiving the leaflet and verbal information. Furthermore, Toumas et al. (Toumas et al., 2009) carried out a study with students to whom they gave a leaflet, and they reported that only 10% of the group performed the technique correctly after reading it. They then gave the students a demonstration and the improvement significantly rose to 62%. These results are similar to the findings reported in TECEPOC and TIEPOC trials.

Although inhalation technique improved at the end of follow-up in the subjects who received the leaflet in the IAR cohort of the TECEPOC study, their performance was very similar to those of the CG. Educational intervention with leaflets alone has been shown to be effective in several studies. Takemura et al. (Takemura et al., 2011; Takemura et al., 2013) found that 39 patients improved adherence to the inhaled therapy, which included the inhalation technique, on the fourth year follow-up visit. Schulte et al. (Schulte et al., 2008) managed to increase the correct inhalation technique percentage by 23%.

However, reading the package leaflet alone is not sufficient to ensure proper inhalation technique (Klijn et al., 2017; Melani, 2021). Many of the package leaflets are often difficult to read, and the print is too small for older patients. In addition, it often contains general rules for handling each device, to comply with legislation, but does not aim to train as a primary objective.

Percentages of improvement in inhalation technique obtained in the present study are lower than those reported in the literature with only some exceptions (Giner et al., 2002; Cabedo García et al., 2010; O’Dwyer et al., 2020). This could be due to the fact that we analysed under the intention to treat principle, whereas the rest of the authors collected the data from the patients who attended the follow-up visit without considering the dropouts.

The teach-back methods with a practical demonstration of inhaler technique with the opportunity for the patients to show how they use their inhaler and receive feedback from instructors is more effective than simple verbal instruction (Klijn et al., 2017). Likewise, as inhaler mastery tends to wane over time, repeated rounds of education and feed-back are required (Axtell et al., 2017; Yoo et al., 2017; Ahn et al., 2019). The problem of this educational approach is that it is time-consuming and seems to remain limited to some successful experiences in real life but does never achieve extensive dissemination (Melani, 2021). Digital technologies could be an improvement, due to their potential to produce devices, such as smart inhalers, with a range of monitoring capabilities, as reported in an interesting review on the subject (Dundon et al., 2020). Applying digital technology advancements to the sector of inhaler technique might offer a large advantage, but the best outcomes will be obtained with a better standardisation of device use and maintenance and strict cooperation among physicians, patients and manufacturers and not working independently (Melani, 2021).

For all these interventions it is critical to evaluate whether patients are able to use their inhaler device correctly. In our study the percentage of incorrect use of inhaler is near 91%. Significant evidence shows that nearly 90% of patients with COPD incorrectly use their inhalers and that many of them display a technique that possibly delivers inadequate doses (Chrystyn et al., 2017; Kocks et al., 2018; Price et al., 2018; Ahn et al., 2019; Duarte-De-Araújo et al., 2019; Melani, 2021; Barnestein-Fonseca et al., 2022) but the percentages vary depending on the checklist used. It could be because there is no exact definition of what is considered a correct inhalation technique. It is not easy to know the operating checklist of use of all marketed inhalers. The observations on a certain drug/inhaler system cannot automatically be extended to another device releasing the same medicine, or to the same device delivering another drug. Moreover, several aspects of inhaler technique and storage remain undefined. Regulatory authorities have strict rules for marketing admission of inhalers, including drug delivery at different flows, positions, and storage conditions, but they cannot be translated to the complexity of real life use (Melani, 2021).

The most frequent errors found in all the devices are the same as those observed in other studies as reflected in the review by Melani A (Melani, 2021). In previous studies, we have found that these errors were related to the patient’s preparation and physical ability to perform the technique, mainly lower peak inhalation flow, lower scores in the MMSE, fewer visits to the pulmonologist, and not having received prior instruction on inhaler use (Barnestein-Fonseca et al., 2013; Barnestein-Fonseca et al., 2022). The errors related to the device are less frequent and related to different flows (coordination in pMDI) and positions (in Turbuhaler^®^) (Chrystyn et al., 2017; Duarte-De-Araújo et al., 2019; Lindh et al., 2019). Despite technology advancements, most subjects do not intuitively achieve inhaler mastery alone (Harb et al., 1902; Melani, 2021). The real-world studies show that an easy-to-use inhaler is not yet available.

Despite the high rate of incorrect technique, many subjects reported having received instruction about the inhalation technique. This could be related to a lack of knowledge of inhaler use and teaching techniques among prescribers (Aksu et al., 2016; Plaza et al., 2018; Al-Otaibi, 2020; Cvetkovski et al., 2020). In addition, it is related to no regular test, reminder and type of instruction (Klijn et al., 2017; Takaku et al., 2017; Kaplan and Price, 2018; Lavorini et al., 2019; Melani, 2021; Lindh et al., 2022). There is extensive literature about self-management education in COPD patients in which different types of educational interventions are checked with a wide spectrum of outcomes (Schrijver et al., 2020). There are not enough interventions focused on inhalation technique training even though there is hard evidence of its usefulness (Klijn et al., 2017). Moreover, the wide majority of studies are centred on patients with asthma, leaving COPD patients aside.

There seems to be agreement about the need that inhalers should be prescribed after a demonstration led by a healthcare professional. Inhalation technique should be performed correctly in every visit to the healthcare centre and supervised by a professional (Aksu et al., 2016; Lavorini et al., 2019; Melani, 2021; Global Iniciative for COPD, 2022). Every time a change in treatment is made, the demonstration by the professional and the patient should be performed (Usmani et al., 2022).

There is little evidence on the appropriate time for reminding patients of inhalation technique. This is partly due to most studies being performed in asthma patients (Bosnic-Anticevich et al., 2010; Takemura et al., 2013; Crane et al., 2014; Axtell et al., 2017; Klijn et al., 2017) although in the last few years some studies enrolled only COPD patients (Bouwmeester et al., 2015; Klijn et al., 2017; Takaku et al., 2017; Yoo et al., 2017; Ahn et al., 2020; Choomuang et al., 2022) but most of the educational programs were too brief.

Three studies scheduled three educational visits at 2-week intervals (Yoo et al., 2017; Kim et al., 2021), or according to a 1-month program (Lee et al., 2016). Takaku et al. showed the effectiveness of education on inhaler technique and adherence for a relatively long period (3 months) after one session of education (Takaku et al., 2017). Another study scheduled three educational visits at 3-month intervals along 6 months and they reported positive results at 3 months (Ahn et al., 2020). We have found similar results, ending up in a recommendation of scheduled reminders each 3 months to improve the inhalation technique in patients with COPD for a longer follow-up (12 months).

Although we have not found any statistical significance, preferences have been defined as modulators of the interventions’ effects in clinical trials, partly due to the opportunity of choosing the treatment based on personal elections which could increase the feeling of self-control related to the learning process, and this would encourage behavioural change, leading to better results (Janevic et al., 2003; Lehmann et al., 2020).

In the preferences’ evaluation it has been suggested that the best method would be to establish the treatment’s efficacy and then use a pragmatic design. In reference to this type of design, a preference trial could be useful in reflecting the usual care from a more realistic point of view (Preference Collaborative Review Group, 2008; Mills et al., 2011). This could be particularly appropriate in health education research, as it is imperative to show the superiority of one of the educational interventions and also to explore the potential effects attributed to the preferences.

Controversial results have been found related to the effect of the preferences (Floyd and Moyer, 2010; Mills et al., 2011; Lehmann et al., 2020). It has also been observed that the preferences can interfere in the recruitment process. In order to avoid this inconvenience, the TECEPOC study was decided to partially randomise patients regarding their preferences, meaning the group allocation already considers the patient’s choice during the recruitment process.

Another aspect to be taken into account with regard to preferences concerns the possibility of modification of the results especially in small sample studies, but no consistency has been observed with regard to the direction of this modification (Floyd and Moyer, 2010; Mills et al., 2011; Lehmann et al., 2020). The present preference study, TECEPOC, has shown that preferences were not related with the efficacy of the designed educational interventions. One possible explanation could be the larger sample size in our case, which means that the preference effect may have disappeared.

These studies have some strengths and limitations. The main strengths are the combination of two studies, with different epidemiological designs, with a big sample size and long follow-up (up to 1 year), which has allowed us to assess the role of patient’s preferences and to know better how often to remind patients of the inhalation technique.

This study also had several limitations. First, the loss of estimation accuracy resulting from the missing data. To diminish this bias, we applied an increase of 40% in the sample size (expected losses) and several phone calls on different days and at different times for unreachable patients and additional appointments for the patients who did not attend the clinic visits. Second, a selection bias could play a role in the results. We got a dropout percentage that was lower than expected but when the similarities between the initial sample and the final sample were analysed, several differences were found. The dropout was more relevant for women, older people and participants with more cognitive impairment. Third, COPD is a chronic progressive illness and the 1 year of follow up could partly explain a higher deterioration in the health outcomes. Another bias, that was taken into account in the analysis of the results, was the rescue mechanism for participants in the control group where the interviewer only corrected the critical mistakes previously agreed by the research team and all interviewers who participated in the study followed the guidelines.

The present study demonstrates the effectiveness of direct training on inhalation technique by a trained professional (e.g. doctor, nurse, pharmacist) with adequate time (e.g. specific medication review consultation) to allow the patient to correct errors through teach-back and repetition. It is an easy intervention to perform, with potentially high effectiveness in real life Although an improvement was observed after the training, there was still a considerable group of patients who were unable to use their device correctly. This would require further analysis of patient characteristics in order to be able to modify some aspects of the training (more frequent reminders), or to assess the need to change inhalers or to use a spacer with some devices.

Further studies are needed to confirm the schedule of reminders and to demonstrate that the intervention can be effectively applied by professionals (doctors, nurses, pharmacists) providing direct clinical care to patients with inhaled medication.

## Data Availability

The original contributions presented in the study are included in the article/supplementary materials, further inquiries can be directed to the corresponding author.
